# Community-based psychosocial support interventions to reduce stigma and improve mental health of people with infectious diseases: a scoping review

**DOI:** 10.1186/s40249-024-01257-6

**Published:** 2024-12-03

**Authors:** Mariska Anindhita, Matsna Haniifah, Arieska Malia Novia Putri, Artasya Karnasih, Feranindhya Agiananda, Finny Fitry Yani, Marinda Asiah Nuril Haya, Trevino Aristaskus Pakasi, Indah Suci Widyahening, Ahmad Fuady, Tom Wingfield

**Affiliations:** 1 Primary Health Care Research and Innovation Center, Indonesia Medical Education and Research Institute, Faculty of Medicine, Universitas Indonesia, Salemba No 6, Jakarta, 10430 Indonesia; 2 Department of Community Medicine, Faculty of Medicine, Universitas Indonesia, Pegangsaan Timur No 16, Jakarta, 10310 Indonesia; 3 Department of Psychiatry, Faculty of Medicine, Universitas Indonesia, Jakarta, Indonesia; 4 Department of Child Health, Faculty of Medicine, Universitas Andalas, Padang, West Sumatera Indonesia; 5 Department of Paediatric, Dr. M. Djamil General Hospital, Padang, West Sumatera Indonesia; 6 Department of Clinical Sciences and International Public Health, Centre for Tuberculosis Research, Liverpool School of Tropical Medicine, Liverpool, UK; 7 Department of Global Public Health, WHO Collaborating Centre on Tuberculosis and Social Medicine, Karolinska Institute, Stockholm, Sweden; 8 Tropical and Infectious Disease Unit, Royal Liverpool and Broadgreen University Hospitals NHS Trust, Liverpool, Liverpool, UK

**Keywords:** Stigma, Tuberculosis, HIV, Leprosy, Community, Depression, Quality of life

## Abstract

**Background:**

Stigma experienced by people with infectious diseases impedes access to care, leading to adverse psychosocial consequences. Community-based interventions could prevent or mitigate these consequences but lack robust evidence. This scoping review aimed to identify and critically appraise community-based psychosocial support interventions to reduce stigma and improve mental health for people affected by stigmatizing infectious diseases including tuberculosis (TB), HIV/AIDS, and leprosy.

**Methods:**

This was a scoping review of literature indexed in PubMed, Web of Science, Elton B. Stephens Company (EBSCO) database, as well as reports in the World Health Organization repository, published from January 2000 to June 2023. We included research articles and reports addressing stigma and mental health disorders among individuals with TB, HIV/AIDS, or leprosy and/or their household members in low- and middle-income and/or high TB burden countries. We extracted information regarding types of psychosocial interventions and their reported impact on health and psychosocial indicators.

**Results:**

Thirty studies were included in this review: 21 (70%) related to HIV/AIDS, seven (23%) leprosy, and two (7%) TB. Of these, eleven were quantitative studies, nine qualitative, and ten mixed-methods. Eleven community-based interventions were reported to reduce infectious disease-related stigma, predominantly internalized and enacted stigma, and improve adherence to medication, quality of life, health-related knowledge, depression symptoms, and psychosocial wellbeing. Most studies involved lay people in the community as supporters of those affected. The predominant reported mechanism of intervention effect was the ability of supporters to enable those affected to feel seen and listened to, to accept their diagnosis, to improve their self-esteem, and to facilitate continuation of their daily lives, and thereby reducing anticipated stigma, self-stigma, and mental illness. Adequate training for lay people was reported to be essential to ensure success of interventions.

**Conclusions:**

This review identified a paucity of high-quality evidence relating to community-based interventions to reduce stigma for infectious diseases. However, such interventions have been reported to reduce stigma and improve mental health among people with HIV/AIDS, leprosy, and TB. Engaging affected communities and peers, through the conception, planning, training, implementation, and evaluation phases, was reported to be essential to optimise intervention uptake, impact, and sustainability.

**Supplementary Information:**

The online version contains supplementary material available at 10.1186/s40249-024-01257-6.

## Background

As of 2023, an estimated 39.9 million people were living with HIV/AIDS, while 10.8 million people became ill with tuberculosis (TB). In the same year, 174,087 new cases of leprosy were reported, marking a 23.8% increase from 2021 [1–3]. People affected by these infectious diseases often face severe stigma and discrimination. While the HIV/AIDS pandemic arose in the early 1980s, historical records show that people afflicted with diseases like leprosy and TB have faced significant stigmatization for centuries [4]. Indeed, the high burden of these diseases is primarily attributed to the interplay of health and socio-economic determinants, such as poverty, limited access to health services, the financial impact associated with seeking healthcare, and notably, pervasive stigma [5–7]. Stigma and discrimination has been defined by World Health Organization (WHO) as ‘a mark of shame, disgrace or disapproval that results in an individual being rejected, discriminated against and excluded from participating in a number of different areas of society’ [8]. Stigma can manifest in various forms, ranging from compulsory identification of people with such diseases by using special clothing or wearing ringing bells when approaching others, to restriction to begging as the only means of subsistence and enforcement of dehumanising segregation measures [9, 10].

These attitudes towards individuals with such diseases are widely recognized as a significant socioeconomic barrier to accessing and engaging in healthcare services [11, 12]. People experiencing symptoms related to TB, HIV/AIDS, and leprosy may hesitate to seek healthcare due to fear or experience of past encounters with stigmatizing attitudes or behaviours from their households, communities, healthcare providers, and even themselves (referred to as self-stigma or internalized stigma) [13, 14]. At the individual level, stigma can lead to care-seeking, diagnostic, and treatment initiation delays, suboptimal treatment outcomes, as well as adverse effects on mental health, such as depression and suicidal thoughts [15–18], and dire socioeconomic consequences [19]. At the household and community levels, the association of stigma with diagnostic delay and lack of engagement with care can lead to an increase in disease transmission, which hinders the efforts of the public health system to end stigmatised infectious diseases in endemic communities [4]. Not only does such stigmatization and discrimination result in mental and physical harm but it is also associated with job losses, reduced educational opportunities for affected groups, and stultification of wider societal and economic development [20].

For the reasons described above, stigma and discrimination represent substantial hurdles to care and prevention of infectious disease globally [21]. Our previous research with 612 people with TB across seven provinces of Indonesia, a high TB burden country, showed that 61% had moderate TB stigma, 41% had depression, and there was a positive correlation between TB stigma and depression levels [22, 23]. Given this catalytic relationship, comprehensive psychosocial support interventions have a critical role to play in mitigating stigma, particularly self-stigma, and associated mental health disorders including depression and anxiety [19, 24, 25]. Indeed, combatting TB stigma was recognised as an essential element of ending TB in the 2018 and 2023 United Nations High Level Meetings on the fight against TB [26, 27].

The importance of addressing stigma has long been a key factor in strategies to eliminate infectious diseases other than TB, including HIV/AIDS and leprosy. The evidence base for the development and evaluation of stigma reduction interventions for people living with or affected by HIV/AIDS has come from both healthcare facility- and community-based studies. Some studies implemented intensive counselling to reduce HIV-related stigma and reported positive results, such as reduced fear amongst people with HIV about disclosing their HIV status, reduced feelings of life limitations due to HIV, and strengthened self-support [24]. For leprosy, community-based interventions using participatory videos and comics have been shown to be effective in increasing knowledge and improving public attitudes towards leprosy, straightforward to replicate in multiple contexts, and not reliant on expensive technology [28]. In addition, counselling in the community, delivered by lay and peer counsellors with appropriate training in effective communication skills, has been shown to reduce leprosy-related internalized stigma [29]. The literature suggests that community-based interventions, which leverage the community as targets, agents, and resources, can help to mitigate health-related stigma [30].

However, in the field of TB, there appears to have been minimal exchange of knowledge or application of learning from strategies to reduce stigma related to HIV and leprosy. While there is evidence for stigma-reduction interventions for people with TB delivered in healthcare facilities [31, 32], focused on positive messaging to the broader community to reduce (mainly enacted) TB stigma [33, 34], and directed towards healthcare workers as recipients [35], there is minimal literature on community-based stigma-reduction activities focused on people with TB and their households. This is important because providing such support in the community could bring it closer to the point of need, thereby increasing its accessibility, impact, and equity. To date, there has been also no single study that has synthesised and critically appraised the evidence on the importance of community-based psychosocial interventions to reduce stigma and learn from the findings across these inter-related infectious diseases: HIV/AIDS, leprosy, and TB [31]. This scoping review is an effort in fill this knowledge gap. Results and recommendations from this study will inform a larger program of research to design, implement, and evaluate a peer-led, community-based psychosocial support intervention for people affected by TB stigma in Indonesia (the TB-CAPS study) [36].

## Methods

This scoping review, which expands on our previous review of the pathways to effectiveness of TB stigma reduction interventions [31], followed internationally recognised methodological standards, including the Arksey and O'Malley guidelines and the PRISMA Scoping Review (PRISMA-ScR) extension list, in order to facilitate an inclusive search strategy that incorporated diverse sources of evidence [37–40].

### Search strategy, inclusion criteria, and exclusion criteria

For this review, we limited the infectious diseases included to TB, HIV/AIDS, and leprosy. These diseases were deliberately chosen due to the strong evidence base that they are all highly stigmatised diseases [4, 21]. The search was conducted for scientific articles recorded in PubMed, Web of Science, and Elton B. Stephens Company (EBSCO), and documents recorded in WHO (https://www.who.int/library/) repository. In this study, we defined community-based interventions as any intervention physically implemented outside of healthcare facilities or healthcare settings and delivered by lay people (e.g., peers, religious leaders, community leaders, young ambassadors), healthcare volunteers, or community healthcare workers. Funding sources of identified interventions were not collated, meaning it is possible that interventions delivered in the community may have been funded by the health system. For the purposes of reviewing community-based psychosocial support, we included several keywords, including but not limited to: counselling, group intervention, social support, emotional support, peer support, support group, home visit, storytelling, psychoeducation, social media, health education, focus group, mobile phone, online, internet, psychosocial support, psychosocial intervention, psychosocial wellbeing, and psychotherapy (see Supplementary materials, Annex A). We included interventions in which the delivery of psychosocial support was one-to-one, in group sessions, in-person, or virtually through a digital platform.

We included articles reporting the above defined community-based psychosocial interventions and their reported impact on outcomes including stigma, mental health disorders (with a focus on depression and anxiety), treatment adherence, treatment outcomes, quality of life, and resilience among people with TB, HIV/AIDS, or leprosy and/or their household members. The interventions were those implemented in low- and middle-income countries and published between 1st January 2000 and 1st June 2023 in English or Bahasa Indonesia. For reviews and meta-analyses, we checked their citations and selected those that fulfilled our eligibility criteria. We excluded editorials, commentaries, and abstracts without full text available.

### Quality assessment of the included literature

The quality of reported studies was assessed qualitatively using the ‘Evidence for Policy and Practice Information and Co-ordinating Centre’ checklist [41]. The tool was chosen for its comparability and, with relation to assessment of study types, its comprehensiveness. We assessed quality based on six quality criteria from these tools including: clear statement of study aims and objectives; robust and appropriate study design; justification of sample size including power calculations where necessary; reliability and validity of outcome measurement scales and tools; statistical analysis plan and reporting; and assessment of bias including amongst others study sample selection. Answers related to these categories were classified as: yes, no, and unclear (see Supplementary materials, Annex B). Quality assessment was done on all studies included as full text reviews in the study in order to identify the potential limitations and contextualise their interpretation.

### Data extraction and analysis

Screened articles from scientific databases and grey literature were independently exported to Covidence systematic review software (Veritas Health Innovation, Melbourne, Australia). We assigned four reviewers (MH, AMN, MAN, AF) to screen the title and abstract, guided by the developed PICO framework (Annex A). Three reviewers (MH, AMN, AF) then screened selected full articles. Any disagreements between reviewers were resolved by discussing between three reviewers to have a consensus to include or exclude the articles for entry into the final analysis.

We extracted relevant data from the selected articles, which was then collated and tabulated into a Microsoft Excel (Microsoft company, Washington, US) database. The summarized data included: study authors, type of articles and study design, country and region of intervention, target population and disease (TB, leprosy, and HIV), type of stigma studied, the tool used to assess stigma, intervention activities, challenges and successes of intervention, and the impact of intervention. Further information on the intervention including format, mode of delivery (by whom, time points, frequency, duration), content, outcomes (both reported and intended if different) and detail on how the intervention reduced or was expected to reduce stigma (theory explicitly stated in the main text or implied in objectives or methods) were also tabulated.

The extracted data were synthesized using quantitative analysis (described in tabular format) and narrative analysis. In narrative analysis, coded findings were grouped into categories to support interpretation and draw meaningful conclusions in accordance with Granheim and Lundman's Content Analysis Method [42]. Following the scoping review protocol, we utilized a Systems Thinking approach to address strategies combating health-related stigma and related mental health disorders. At the end of the review, we developed a new framework by combining two previously developed frameworks, which are the Health Stigma and Discrimination Framework and Nuttall and Fuady's framework [31, 38], then displayed the reported or proven and the unproven hypothesised mechanisms and impact of interventions on reducing stigma and improving mental health in the framework.

## Results

### Characteristics of included studies

**Fig. 1:**
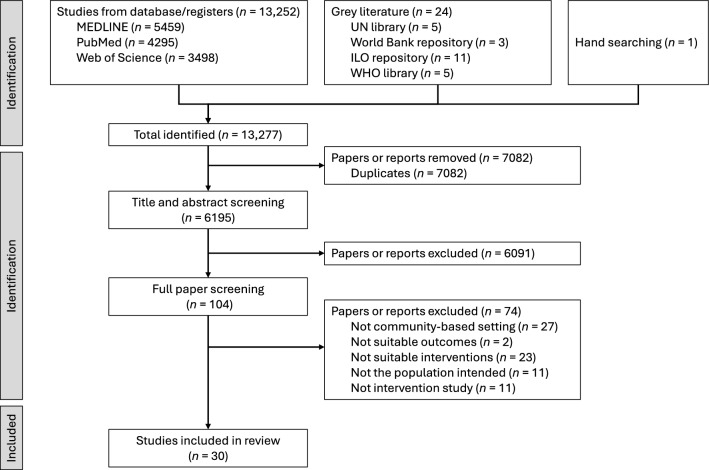
PRISMA-ScR study flow chart

We initially identified 13,252 studies, and—after title, abstract, and full text screening—30 articles (Fig. 1) were included in the analysis: 21 (70%) related to HIV/AIDS, seven (23%) leprosy, and two (7%) TB. Of the 30 articles, most (*n* = 20, 67%) were from Africa, nine (30%) from Asia, and one (3%) from South America. Prominently featured countries were South Africa (*n* = 8, 26%), Indonesia (*n* = 5, 16%), Zimbabwe (*n* = 3, 10%), and Ethiopia (*n* = 3, 10%). Eleven studies were quantitative, nine were qualitative, and ten were mixed methods (Table 1). Twenty-eight studies were conducted in community, one study in a school, and one study in the community-based premises of a civil society organization.

**Table 1:** Study designs and settings of reviewed studies

Analysis method	Number of studies	Setting			Infectious disease		
		Community	Service office	School	HIV	Leprosy	Tuberculosis
Quantitative	11	10	0	1	9	1	1
Qualitative	9	8	1	0	9	0	0
Mixed-methods	10	10	0	0	3	6	1

### Quality of the included studies

In our qualitative assessment of the quality of included studies, we found most of the studies (*n* = 20/30) (see Annex B) were not randomized controlled trials and therefore prone to biases in sample selection. Six of the studies mentioned sample size but without sufficient explanation or reported calculations [24, 43–47].

### Intervention providers

This review found four types of intervention providers: peers (*n* = 13) [24, 47–58], community members (*n* = 5) [34, 59–62], combination of peers and community members (*n* = 10) [28, 29, 43, 46, 63–68], and community healthcare workers (*n* = 2) [44, 45]. Peers (herein termed “Peer Supporters”) are defined as individuals sharing common characteristics or experiences, specifically lived experiences related to a certain disease, with the individuals they support [51]. TB Clubs, for example, invited people with TB and TB survivors to become peer supporters in their communities [50]. Six studies recruited individuals (not people with direct lived experience of the disease, such as survivors) who had influence within their communities, such as youth ambassadors [54], Community Popular Opinion Leaders [58], lay grandmothers [61], female health educators [49], community volunteers [34], and mentor mothers [47], to provide the interventions. Eleven articles from six studies recruited individuals from both the community of people with the disease and non-affected community members, such as their families [46, 67], local disability organization staff [29, 43, 65], or general community members [29, 34, 63, 64, 66, 68] to improve collaboration between implementers and recipients of the intervention. Such recruited individuals take on roles as peer supporters with titles as volunteers, facilitators, mentors, counsellors, leaders, and mobilizers within the context of the interventions.

### Training and module training for intervention providers

Where interventions were delivered by peer supporters, most (9/13 articles) were trained by either international training institutions [24] or by researchers [48, 56, 57] using a training curriculum/module prepared prior to the study implementation. Such modules included Médecins Sans Frontières and UNICEF’s curricula [56] and the Dennis Peer Support Model for training against HIV stigma [48]. Six articles mentioned the module content focused on several key areas, including (a) understanding and addressing disease-related stigma, (b) developing skills to give presentations and facilitate group discussions, (c) planning and implementing community-based projects [46, 58, 67], and (d) elements of cognitive behavioural therapy combined with human rights-based counselling related to stigma and discrimination [29, 43, 65]. Four articles [47, 50–52] did not report whether peers were trained prior to their study implementation.

### Types of community-based interventions

We identified 12 types of psychosocial interventions: group counselling [24, 44, 48, 50, 57, 63, 66], individual counselling [24, 29, 43, 65], family counselling [29, 43, 62, 65], escort to healthcare facilities and social support [51], home visits [47, 53, 55, 56], formation of youth volunteers [59], community conversation/participation [59, 60], mass health education [34], mass media campaign [69], mass health education [34], religious activities [59], and media-assisted counselling [28, 29, 43, 48, 54, 65] (Table 2).

**Table 2:** Pathways to impact of psychosocial interventions for people affected by tuberculosis, HIV/AIDS, and leprosy

Activities	Description	Outcomes	Challenges of the intervention	Reported pathways and impact of the intervention to reduce stigma
Group counselling	People with HIV [24, 48, 57, 63, 66], tuberculosis (TB) [50], or leprosy [44] met in group counselling session to identify and share their stressful thoughts regarding shame, disease disclosure and other disease-related issues. The group met frequently facilitated by either a trained facilitator or within a self-help group	Improvement in self or internalized stigma [24, 57, 66], reduced drop-out rate in TB treatment [50], and increased communication about sexual health [48]	Difficulty in recruiting and retaining lay or peer facilitators due to conflicts in schedules and commitments, and issues with remuneration	Group counselling sessions, led by peers or affected communities in regular sessions help to empower people with HIV, TB, and leprosy, enabling acceptance of their diagnosis and improvements in adherence to treatment
Individual counselling	People with HIV and leprosy [24, 29, 43, 65] met in person with an individual trained facilitator to identify and discuss their stressful thoughts regarding shame, disease disclosure and other disease-related issues. The counselling was applied following diagnosis but prior to starting treatment [61]	Reducing internalized stigma [43] and anticipated stigma [65], empowering people affected to be more confident and aware of their rights [43], and reducing depression [24]	Not reported	Multi-session individual counselling reduced stigma and depression by improving people’s confidence [43]
Family counselling	Trained lay persons provided counselling to people with disease and their families [29, 43, 62, 65] to facilitate family discussions and problem-solving	Improving resilience and quality of life of people affected [62], reducing internalized stigma [43] and anticipated stigma [65], and improving confidence and awareness of rights as citizens, including right to health and healthcare [29]	Not reported	Family counselling can promote resilience and support from the family unit. Through this supportive relationship, people with HIV and leprosy can develop their self-esteem and self-efficacy, reducing internalized stigma and increase QoL [62]
Escort to healthcare facilities and social support	Trained lay persons accompany people who inject drugs to facilitate and coordinate health care services and social support, including those without HIV and ancillary services for those who have HIV [51]	Improving adherence to treatment and engagement with health care services such as HIV testing. Enhancing confidence and motivation and emotional support. Reducing enacted stigma from healthcare workers at facilities	Supporters often did multiple roles and set the target on the number of clients they supported each month, which was reported to sometimes negatively affect the quality of the support (i.e., inverse relationship between number of clients and quality of support)	The escort provided emotional support and their presence in accessing health and social care services acted as a buffer from or intermediary between healthcare workers, thus reducing enacted stigma from healthcare workers towards those affected. It led to enhanced confidence and motivation to seek health care services and promoted adherence to treatment
Home visit	Lay people make a visit to the house of people affected by the disease to check their adherence to management and provide support and knowledge during their treatment [47, 53, 55, 56]	Reducing stigma [53, 56] and sense of alienation and isolation, along with increasing QoL mainly among people with advanced HIV disease [47]	In some cases, the home visit resulted in inadvertent disclosure or revealing of disease to neighbours or others in the community thus causing further stigma related to the disease	Home visit by the trained lay person other than providing emotional and social support, helps people living with HIV/AIDS (PLWHA) to socialize in public and challenge other people who show stigma to PLWHA. This was reported to contribute to a reduction in stigma and an increase in QoL
Formation of youth volunteers	Forming a youth group to support dissemination of information about disease to the community. Trained to play their role as key ‘change agents’ for the prevention of disease in their communities [59]	Improve HIV knowledge and reduce community stigma	This activity depended on the dedication and active participation of the researchers acting as facilitators. Funding would be necessary to support the facilitators' efforts	The volunteers act as ‘agents of change’ and have roles in delivering knowledge about HIV/AIDS to their community in local, public spaces. This resulted in improved knowledge of HIV/AIDS and reduced level of community stigma
Community conversation/participation	Discussions [60] and work together [59] among local people, guided by a trained facilitator, with the intention to support critical thinking and problem solving from within the community around key pertinent health issues	Increasing awareness and knowledge about HIV/AIDS, and reducing HIV/AIDS stigma [59, 60]	Recruitment and commitment from the participants in the community for continued involvement in the activities was challenging Facilitators must be someone whom the participants respect and are inspired by in order for participants to be able to fully engage and develop effective community-led solutions	There was a broader interaction between PLWHA and other community members, thus increasing the chance to share the reality of HIV and reduce the stigma surrounding HIV. Given this is an intervention or programme that the community develops itself, this nurtures a sense of both ownership and common purpose among the participants to support the success of HIV care and prevention
Mass media campaign (Current form: PodCast, YouTube)	Campaign through weekly sessions on radio stations [69] of “Radio Diaries” (RD) programme on HIV-related stigma or RD + group discussion, featuring two people with the disease who narrated 10-min segments about issues and key events in their lives, such as experiences with health services	Reducing self-stigma and feelings of fear and shame on Radio Diaries group	The materials given to participants were different between genders to ensure gender sensitive and responsive but variations in the quality of the received materials were reported	The PLWHA who shared their stories through the program reduced their shame of having HIV, which in turn mitigated the radio audience’s fear of contact with PLWHA. Thus, the radio program was reported to reduce internalized and externalized stigma
Mass health education, with additional poster and pamphlet	Community volunteers organized health education to the public, one-on-one discussion, educational pamphlets and posters, and street rallies [34]	Increasing TB knowledge and attitude; reducing anticipated stigma towards people with TB	Insufficient and low quality of training. Community volunteers were not trained health workers, received an excessive new knowledge during the 2-day training and may not have fully understood the cause, transmission, signs, and cure of TB	Failed to improve TB knowledge and attitudes, and misconceptions, including stigmatizing attitudes towards people with TB remained.
Establishing a community learning centre	The volunteers used a specific area of the community library to form an HIV/AIDS information center [59], using documents from the community hospital, provincial health office and the National Scientific Conference	Increasing knowledge about HIV/AIDS and developing concrete and practical action plans for improving community awareness	Lack of interaction with the information center over time if no specific innovation or outreach. Such centers should use adjunctive methods to sustain the motivation of the community to learn about HIV/AIDS	Disseminating news and knowledge on HIV/AIDS disease at the community library increased the levels of accurate HIV/AIDS knowledge in the intervention village thus reducing stigma
Religious activities	Religious activities were conducted to release suffering [59]. Participants attended a sermon, then discussed their suffering in different situations, noting the similarity of suffering between PLWHA and others	Reduce external and self-stigma	Needed commitment and involvement -specifically of the religious leaders—in the community	Discussions during religious activities increases the sense of similarity between people with and without the disease thus promoting empathy and moral support, which results in reduced stigma
Media-assisted counselling	Counselling session using tailored cartoon or comic strips [28, 29, 43, 54, 65]. Participants were invited to make books and draw to express their experiences. Furthermore, participants were asked to make comics which were then displayed to public. Counselling session also conducted via a mobile phone to link people living with HIV with their peers and nurses [48]	Increasing knowledge and removing misconceptions amongst the public about the leprosy and HIV diseases. Improving self-efficacy. Encouragement to know one’s HIV status and promote healthy living	The intervention did not require expensive technology, but some costs and time were involved	The media, featuring content created by people with the disease, serves as an indirect means of introducing their condition to the public, resulting in heightened awareness and knowledge about the disease among the public. Through creating art about their disease and their journey, a sense of self-esteem and self-efficacy can be promoted amongst affected people

*TB* tuberculosis; *HIV/AIDS* Human immunodeficiency virus/acquired immune deficiency syndrome; *PLWHA* People living with HIV/AIDS; *RD* Radio diaries; *QoL* Quality of life; *WHO* World Health Organization

### Impact of community-based interventions

The identified psychosocial interventions measured and reported a variety of outcomes dependent on the objective of the studies, ranging from increasing knowledge about the diseases to improving quality of life (see Table 2). Following the Health Stigma and Discrimination framework, we grouped the impact of interventions into controlling drivers and facilitators of stigma, reducing manifestation of stigma, and improving other outcomes.

Activities to control drivers and facilitators of stigma predominantly included education in the community, which aimed to reduce stereotypes and social judgement as well as to improve social cohesion [70]. Community conversation/participation allowed the community to brainstorm their own solution regarding stigma in HIV and grew sense of common purpose to prevent HIV [59, 60]. This intervention enabled community members to live side by side with people with HIV and actively contribute to reducing disease-related stigma. One study highlighted that establishing community participation in the intervention could help sustainability by ensuring people in the community to become the agents of change [59].

Activities such as mass media campaigns were reported to have positive effects on the community by increasing the general public’s knowledge, reducing their fear about the diseases, and helping reduce stigma manifestation towards people with the diseases. In HIV, the campaign increased the self-confidence of people with HIV and empower them to disclose their status [64]. However, a mass health education and public rally was reported to not increase knowledge and attitude and to not defray misconceptions towards TB [34]. Among reported challenges were insufficient training for the volunteers and incomplete information conveyed through posters and pamphlets [34].

These interventions at the community level, together with psychosocial support from Peer Supporters, contributed to the manifestation control of stigma by reducing perceived or anticipated stigma and secondary stigma—which is experienced by the families and friends of people with diseases [62]. Peer supporters often had expanded roles by providing counselling and companionship to access HIV care, which in one case allowed them to act as a facilitator between people injecting drugs and their healthcare providers [51]. Counselling, either in group or individual settings, was reported to empower people with diseases and increase their self-confidence, leading to a reduction of internalized stigma [43, 62]. Interventions focused on reducing internalized stigma also showed positive impacts by creating a sense of freedom, thereby mitigating fear of disclosing disease status, improving self-efficacy, and reducing depression [24, 29, 45, 47, 50, 52, 54, 55, 58, 61, 67, 69]. Through reducing the manifestation of stigma, other outcomes were reported to be improved, such as treatment adherence and completion, ensuring access to health care, increasing quality of life, and reducing feelings of isolation [50].

### Outcome assessment of the interventions

We found and grouped outcomes into seven: reducing stigma, reducing depression, improving adherence/compliance with treatment, improving quality of life, improving self-efficacy (improving an individual's confidence in their ability to set achievable goals, seek feedback, and model successful behaviour [71]), improving knowledge, and improving psychosocial wellbeing (a sense of wellbeing that include the satisfaction in life and balance positive and negative affect of individual [63]). These seven outcomes were evaluated using several scales/tools even when the outcome being measured was the same, including nine separate tools to measure HIV-related stigma, and it was notable that most of the tools used were not locally validated prior to the study data collection (Table 3).

**Table 3:** Tools used to measure the outcomes of interventions in the reviewed studies

Outcomes	Analysis method	Scale/tool	Internal and external validation prior the study	Time of evaluation
Reducing stigma	Quantitative	The Explanatory Model Interview Catalogue Community Stigma Scale (EMIC-CSS) [28, 65]	Yes	Before the intervention and after the intervention at 3-month follow-up [28]
		Social Distance Scale (SDS) [28, 65]	Yes	Baseline and final of intervention [65]
		6-Question Questionnaire [28]	Yes	
		HIV/AIDS stigma questionnaire [59]	Information not given	Before and at the end of the intervention
		The HIV/AIDS stigma instrument—PLWH (HASI-P) [46]	Yes	Before and after intervention
		AIDS-related stigma measure (for community) [46]	Yes	Before and after intervention
		Structured questionnaire [50]	Information not given	Information not given
		Internalized AIDS-Related Stigma Scale (IA-RSS) [24]	Yes	Information not given
		The Berger HIV Stigma Scale [53, 57]	Yes	Baseline during pregnancy and 4 months postpartum (mothers), at birth (infants) [57]. Baseline and after intervention [53]
		SARI stigma scale [43, 65]	Yes	Baseline and final survey [65] Baseline, during, and after intervention [43]
		Participation Scale Short [29, 65]	Yes	Baseline and final intervention [65]
		WMM Cultural Stigma Scale for WLHIV in Botswana [57]	Yes	Baseline during pregnancy and 4 months postpartum (mothers), at birth (infants)
		Nyblade and MacQuarrie stigma scales [69]	Yes	Information not given
		Discrimination and Stigma Scale (DISC-12) [45]	Yes	Before, 3 and 12 months after intervention
		The Internationalized Stigma in Mental Illness (ISMI) scale [45]	Yes	
	Qualitative	Self-developed questionnaire [29, 47, 49, 58]		Baseline, after activities and final intervention [29] No [47] Baseline and 3 months after intervention [49] Baseline, 12- and 24-month follow-up [58]
		FGD/Interview guides [24, 28, 29, 50, 51, 60, 61, 65, 67, 68]	Yes [51, 56] No [24, 28, 50, 60, 61]	Baseline, after activities and final intervention [29] Baseline and final intervention [65] No [24, 28, 50, 60, 61]
		Reports and field note, Naïve sketches: participants’ notes and Weekly reports	Information not given	Throughout and after the intervention [67] Baseline, after activities and final intervention [29] After each day and at the end of the intervention [68]
		Participatory observations [59]	Information not given	Before and at the end of the intervention
Improve adherence/compliance	Quantitative	Usability parameters of digital platform [52]	Information not given	Baseline, mid-term and 3 months after intervention
		Critical adherence behaviours (differential scale by Velasquez) [52]		
		Structured questionnaire [52]		
	Qualitative	Reports and field note, Naïve sketches: participants’ notes and Weekly reports [67]	Information not given	Throughout and after the intervention
		Interview guides [61]	Information not given	Information not given
		Open-ended questions [50]	Information not given	Information not given
Improve QoL	Quantitative	WHOQOL-HIVBREF [43, 55, 62, 65]	Yes	At baseline and every 4 months during the intervention
		The Internal AIDS-Related stigma questionnaire [55]	Yes	At baseline and every P﻿lease c﻿hange to: 4 months on the intervention
		The WHO DAS 2.0 [45, 57]	Yes	Baseline during pregnancy and 4 months postpartum (mothers), at birth (infants) [57] Before, 3 and 12 months after intervention [45]
		The Dermatology Life Quality Index [45]	Yes	Before, 3 and 12 months after intervention
		The HIV/AIDS Targeted Quality of Life Scale [24]	Yes	Information not given
		The Connor-Davidson Resilience Scale (CD-RISC) [62]	Yes	The baseline, the first follow up and the second follow up
		The Dermatology Life Quality Index [44]	Yes	Baseline and at the 3-month follow up
		The WHO Disability Assessment Schedule (WHODAS) 2.0 [44]		
		Questionnaire focused on socio-economic characteristics [44]		
	Qualitative	Interview notes and feedback [62]	Yes	At baseline, first follow up and second follow-up
Improving self-efficacy	Quantitative	The Rosenberg Self-Esteem Scale [54]	Yes	Information not given
		The Self-Efficacy Questionnaire for Children [54]		
		The Strengths and Difficulties Questionnaire [54]		
		The coping self-efficacy scale and the spirituality wellbeing scale [63]	Yes	Beginning and four repetitive post-test three months apart
		The Patient-Reported Outcomes Measurement Information System (PROMIS) Ability to Participate in Social Roles and Activities Short Form scale [57]	Yes	Baseline during pregnancy and 4 months postpartum (mothers), at birth (infants)
	Qualitative	Interview guide [48]	Information not given	Information not given
Improving psychosocial wellbeing	Quantitative	The mental health continuum short-form scale [63]	Yes	Beginning and four repetitive post-test three months apart
		The patient health questionnaire/PHQ-9 [45, 63]	Yes	
		The satisfaction with life scale [63]	Yes	
		Six different domains of social support [57]	Yes	
		The Oslo Social Support Scale [45]	Yes	Before, 3 and 12 months after intervention
Improving knowledge	Quantitative	HIV related knowledge with Heckman 12 item scale [49]	Yes	The baseline and 3 months after the intervention
		Attitudes scale [49]		
		Rosenberg self-esteem scale [49]		
		Questionnaire with 5 items [59]	Information not given	Before and at the end of the intervention
		An adapted WHO Knowledge, Attitude, and Practices questionnaire [34]	Information not given	Pre-intervention and repeated at 6 months post-intervention
		6-Question Questionnaire [28]	Yes	Before, immediately after, and 3 months after the intervention
	Qualitative	Reports and field note, Naïve sketches: participants' notes, and Weekly reports [67]	Information not given	Throughout and after the intervention
		FGD/Interview guide [28, 48, 59]	Information not given	Before and at the end of the intervention [59]
		Participatory observations [59]	Information not given	Before and at the end of the intervention
Reducing depression	Quantitative	The Child Depression Inventory [51]	Yes	Information not given
		The Center for Epidemiologic Studies Depression Scale (CES-D) [24, 55, 57]	Yes	Information not given [24] Baseline during pregnancy and 4 months postpartum (mothers), at birth (infants) [57] The baseline and 3 months after the intervention [55]
		The Post-Traumatic Checklist for DSM-5 [57]	Yes	Baseline during pregnancy and 4 months postpartum (mothers), at birth (infants)
	Qualitative	Interview guide [48]	Information not given	Information not given
		FGD and interview guides [24]	Information not given	Information not given

*CES-D* Center for Epidemiologic Studies Depression Scale; *CD-RISC* Connor-Davidson Resilience Scale; *EMIC-CSS* Explanatory Model Interview Catalogue Community Stigma Scale; *FGD* focus group discussion; *HIV/AIDS* Human Immunodeficiency Virus/acquired immune deficiency syndrome; *IA-RSS* Internalized AIDS-Related Stigma Scale; *ISMI* Internationalized Stigma in Mental Illness; *PHQ* patient health questionnaire *PLWHA* People living with HIV/AIDS; *PROMIS* Patient-Reported Outcomes Measurement Information System; *RD* Radio diaries*; TB* tuberculosis; *WHO* World Health Organization; *WHODAS* WHO Disability Assessment Schedule

Twenty-one studies applied quantitative methods [24, 28, 29, 34, 45, 49–53, 55, 57, 59–63, 65, 67–69], of which 16 used structured questionnaires and performed internal/external validation. Nine studies applied qualitative methods by conducting interviews and focus group discussions (FGDs) with key community members [24, 28, 29, 50, 51, 60, 61, 65, 67, 68], including religious leaders, and reflection notes—written by clients or participants—were used [43, 59] to provide insights and perspectives that can further enrich the understanding of the intervention’s impact and the experiences of those receiving or engaging with the intervention. In addition, mixed methods research techniques were applied in ten studies by combining surveys (using structured questionnaires or quantitative assessment prior to, during, and following intervention implementation), interviews (semi-structured interview, in-depth interview, KIIs, and mixture), FGDs and participatory observation.

## Discussion

**Fig. 2:**
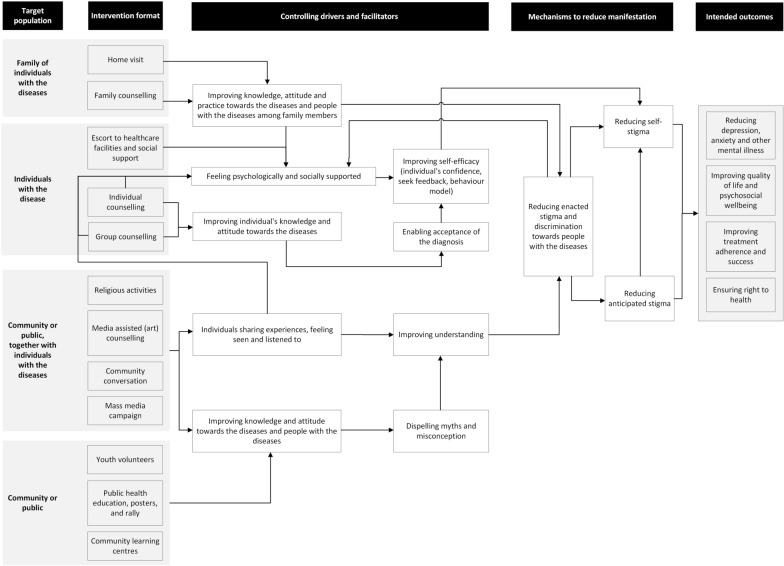
Intervention activities, mechanisms to reduce manifestations, and intended outcomes of the interventions, mapped onto an integrated framework for stigma reduction in infectious diseases [26, 32]

This scoping review highlights some proven and hypothesised mechanisms and impacts of implementing community-based psychosocial interventions for people with infectious diseases including TB, HIV/AIDS, and leprosy. The reviewed studies show that the provision of psychosocial support was not only reported to reduce stigma but also improved the recipients’ knowledge about their disease, mental health, quality of life, and treatment adherence (Fig. 2). This review showed that multiple tools and scales have been used to measure stigma. Due to their intersection, stigma-reduction interventions need to also include the evaluation of depressive symptoms, treatment adherence, quality of life, self-efficacy, and psychosocial well-being. This review also highlights that peers and other community members have the potential to deliver community-based stigma-reduction interventions as facilitators through both individual and group counselling modalities. In addition, they can provide companionship and act as a source of information during disease treatment to reduce self-stigma. At the community level, community members can act to deliver accurate, appropriate information and correct myths and misperceptions in their community around specific infectious diseases thereby potentially reducing enacted stigma. However, caution must be taken given the evidence that some TB health education campaigns have been unable to deliver accurate public health messages and been associated with persistent misconceptions about TB [34].

This review suggests that group counselling, the most applied community-based psychosocial intervention for people with infectious diseases, is useful to share experiences of TB but is best delivered using a person-centred approach that does not compromise privacy [56]. To do this, the group leaders, facilitators, and members need to ensure a safe environment that allows people affected by stigmatising infectious diseases to comfortably share their feelings, experiences, and testimonies [24]. Ensuring a safe and judgement-free environment is critical in group settings to prevent further enacted stigma. Therefore, peer facilitators must obtain participant consent, establish clear confidentiality guidelines, and consistently reinforce the importance of privacy throughout the sessions [72]. This will fortify group formation, reduce feelings of isolation, and could contribute towards reducing self-stigmatisation. Group meetings involving family members may be useful to facilitate evaluation and mitigation of stigma within the family or household, including through provision of psychological, financial, and physical care support by family members for the affected person. Such family support and input, shown in the evidence identified to help people with HIV/AIDS and mental health [18, 73, 74], can also enable better TB treatment adherence and completion [75].

Peer supporters were the most frequently mentioned implementers of stigma-reduction interventions in the studies identified. Involving peer supporters in stigma-reduction interventions is believed to enhance connection amongst people affected by a shared experience of disease-related stigma, thereby contributing to a reduction in internalized or self-stigma and improving broader outcomes [76] However, achieving this successfully requires sufficient training, both in terms of quality and content [32, 34]. A cross-cutting intervention activity that was reported to be crucial for developing communication and empathy skills amongst peers, and thereby optimising intervention impact, was suitable training prior to the intervention implementation. Dennis peer support training, for example, facilitate colleagues to understand support models, the role of peer mentors, building good relationships. This training equipped peers to communicate well while providing peer support, ensure confidentiality between peer mentors, mentees, and other team members. In addition, this training provided an understanding of intersectional stigma and its impact, knowledge about self-efficacy, and technical guidance for interventions [48]. During training, peers can learn to manage group dynamics, including managing emotions, to understand accompaniment boundaries, and to develop skills as group facilitator [77]. Therefore, the training requires a well-planned comprehensive training module, which can be tailored according to peers’ needs.

Stigma reduction activities and interventions also need to be more inclusive between genders where possible. While female volunteers often play a crucial role in providing supports [47], it was notable involvement of male volunteers in psychosocial support activities was limited despite male constituting the majority of TB and leprosy cases [78, 79]. The gender-responsive activities can improve outcomes in men, an area that merits further attention.

Evaluating interventions is crucial, not only to determine their effectiveness but also to assess their acceptability, sustainability, and replicability in diverse settings. Using validated quantitative instruments is crucial to ensure the reliability, accuracy, and fairness of the measurements across diverse linguistic and cultural groups [80, 81]. In addition to pre- and post-test quantitative evaluation, complementary qualitative assessment is required to explore the achievement, challenges, obstacles, and opportunities for scaling up the intervention. A reflection note, in particular, can enhance the overall assessment of the intervention by allowing all involved actors to reflect on their learning, process feedback, and determine the knowledge and skills they learned, thereby deepening their understanding and promoting self-awareness [82]. However, it is worth noting the diversity of often unvalidated tools to measure the intervention outcomes that were identified in this scoping review, which limits replicability and generalizability.

This review highlights that co-creation and co-design of interventions with affected communities, especially those related to reducing stigma related to infectious diseases, is a critical step. Involving affected communities in the intervention at all stages not only puts such communities at the centre of the study, research, or program, but can also be empowering and increase their capacity to take ownership of interventions and establish collective grassroots actions and strategies to overcome disease-related stigma. The community involvement ranges from forming a forum to identify problems and the root causes of stigma, as well as to evaluate whether stigma was a societal issue [59, 60], to encouraging people from affected communities to become facilitators and mentors for people with the diseases [47, 49, 61].

In addition, involving community in co-developing an intervention can promote understanding of its applicability and sustainability in public health setting, including how to embed the intervention within the existing health system. This process can be challenging with several obstacles encountered to achieve such integration. For example, there may be individual, infrastructural or system-level resistance to change, difficulty in adapting knowledge and techniques to local contexts, lack of infrastructure, and methods complexity (or dearth of “how to” practical guidance) that can perpetuate a gap between research findings and their implementation in real-world healthcare settings [83]. Developing trust and common understanding between researchers and community may resolve these problems [84, 85].

This review has several limitations. First, the development and implementation of community-based interventions are affected by local sociocultural context that may not be suitable to be implemented in other areas. For example, religious activities in a church and community conversation need to consider community cohesion and religiosity that may not be applicable in other local settings and therefore has limited generalization. There was also a geographical imbalance, with most studies being conducted in Africa. Given the high HIV prevalence in such African countries, more studies were conducted to address HIV-related stigma problems, and the cultural aspects were appropriate for community-based interventions. It may also have been influenced by the screening strategy, which included only documents in English and Bahasa, and excluded articles in other common languages such as French, Spanish, Chinese, and Arabic. Additionally, some studies utilized assessment tools that were not validated for the specific contexts in which they were implemented, which could compromise the accuracy and reliability of the reported outcomes.

## Conclusions

This review identified a paucity of high-quality evidence relating to community-based interventions to reduce stigma for infectious diseases with more evidence in the fields of HIV/AIDS and leprosy than TB. However, the limited studies identified highlighted the importance of involving peers and community members in the conception, design, delivery, and evaluation of community-based psychosocial interventions for people affected by infectious diseases to reduce stigma and improve mental health, quality of life and treatment adherence. Involvement of peers and community members is essential not only during implementation of the intervention but throughout all stages from conception through co-development to analysis and dissemination in order to ensure intervention acceptability and long-term sustainability.

## Supplementary Information

Supplementary Material 1.

## Data Availability

The data of this study are available by requesting to corresponding author.

## Abbreviations

FGD: Focused group discussion
HIV/AIDS: Human immunodeficiency virus/acquired immune deficiency syndrome
PICO: Population-intervention-control-outcome
PRISMA-ScR: Preferred reporting items for systematic reviews and meta-analyses for scoping review
TB: Tuberculosis
UNICEF: United Nations Children’s Fund
WHO: World Health Organization
