# Supplementary material for: Community-based psychosocial support interventions to reduce stigma and improve mental health of people with infectious diseases: a scoping review
Source: Infect Dis Poverty. 2024 Dec 3;13:90. doi: 10.1186/s40249-024-01257-6 (PMC11613911; doi:10.1186/s40249-024-01257-6)
Supplement: Supplementary file 1 — Supplementary Material 1. [file 40249_2024_1257_MOESM1_ESM.docx]

**Annex**

**Community-based psychosocial support to reduce stigma related to infectious diseases: a scoping review**

**Annex A.** PICO Framework for searching and screening

| **Criteria** | **Determinants** |
| --- | --- |
| Population | 1. People with TB, HIV/AIDS, or leprosy  - tubercul* , TB - hiv* , human immunodeficicency virus, AIDS - lepros* , Hansen* disease  1. Living in countries classified as low- and middle-income country:  - Developing Countries - Afghanistan OR Albania OR Algeria OR Angola OR Antigua OR Barbuda OR Argentina OR Armenia OR Armenian OR Aruba OR Azerbaijan OR Bahrain OR Bangladesh OR Barbados OR Benin OR Byelarus OR Byelorussian OR Belarus OR Belorussian OR Belorussia OR Belize OR Bhutan OR Bolivia OR Bosnia OR Herzegovina OR Hercegovina OR Botswana OR Brasil OR Brazil OR Bulgaria OR “Burkina Faso” OR “Burkina Fasso” OR “Upper Volta” OR Burundi OR Urundi OR Cambodia OR “Khmer Republic” OR Kampuchea OR Cameroon OR Cameroons OR Cameron OR Camerons OR “Cape Verde” OR “Central African Republic” OR Chad OR Chile OR China OR Colombia OR Comoros OR “Comoro Islands” OR Comores OR Mayotte OR Congo OR Zaire OR “Costa Rica” OR “Cote d'Ivoire” OR “Ivory Coast” OR Croatia OR Cuba OR Cyprus OR Czechoslovakia OR Czech Republic OR Slovakia OR “Slovak Republic” OR Djibouti OR “French Somaliland” OR Dominica OR “Dominican Republic” OR “East Timor” OR “East Timur” OR “Timor Leste” OR Ecuador OR Egypt OR “United Arab Republic” OR “El Salvador” OR Eritrea OR Estonia OR Ethiopia OR Fiji OR Gabon OR Gabonese Republic OR Gambia OR Gaza OR “Georgia Republic” OR “Georgian Republic” OR Ghana OR “Gold Coast” OR Greece OR Grenada OR Guatemala OR Guinea OR Guam OR Guiana OR Guyana OR Haiti OR Honduras OR Hungary OR India OR Maldives OR Indonesia OR Iran OR Iraq OR “Isle of Man” OR Jamaica OR Jordan OR Kazakhstan OR Kazakh OR Kenya OR Kiribati OR Korea OR Kosovo OR Kyrgyzstan OR Kirghizia OR “Kyrgyz Republic” OR Kirghiz OR Kirgizstan OR “Lao PDR” OR Laos OR Latvia OR Lebanon OR Lesotho OR Basutoland OR Liberia OR Libya OR Lithuania OR Macedonia OR Madagascar OR “Malagasy Republic” OR Malaysia OR Malaya OR Malay OR Sabah OR Sarawak OR Malawi OR Nyasaland OR Mali OR Malta OR “Marshall Islands” OR Mauritania OR Mauritius OR “Agalega Islands” OR Mexico OR Micronesia OR “Middle East” OR Moldova OR Moldovia OR Moldovian OR Mongolia OR Montenegro OR Morocco OR Ifni OR Mozambique OR Myanmar OR Myanma OR Burma OR Namibia OR Nepal OR “Netherlands Antilles” OR “New Caledonia” OR Nicaragua OR Niger OR Nigeria OR “Northern Mariana Islands” OR Oman OR Muscat OR Pakistan OR Palau OR Palestine OR Panama OR Paraguay OR Peru OR Philippines OR Philipines OR Phillipines OR Phillippines OR Poland OR Portugal OR Puerto Rico OR Romania OR Rumania OR Roumania OR Russia OR Russian OR Rwanda OR Ruanda OR “Saint Kitts” OR “St Kitts” OR Nevis OR “Saint Lucia” OR “St Lucia” OR “Saint Vincent” OR “St Vincent” OR Grenadines OR Samoa OR “Samoan Islands” OR “Navigator Island” OR “Navigator Islands” OR “Sao Tome” OR “Saudi Arabia” OR Senegal OR Serbia OR Montenegro OR Seychelles OR “Sierra Leone” OR Slovenia OR “Sri Lanka” OR Ceylon OR “Solomon Islands” OR Somalia OR “South Africa” OR Sudan OR Suriname OR Surinam OR Swaziland OR Syria OR Tajikistan OR Tadzhikistan OR Tadjikistan OR Tadzhik OR Tanzania OR Thailand OR Togo OR “Togolese Republic” OR Tonga OR Trinidad OR Tobago OR Tunisia OR Turkey OR Turkmenistan OR Turkmen OR Uganda OR Ukraine OR Uruguay OR USSR OR “Soviet Union” OR “Union of Soviet Socialist Republics” OR Uzbekistan OR Uzbek OR Vanuatu OR “New Hebrides” OR Venezuela OR Vietnam OR “Viet Nam” OR “West Bank” OR Yemen OR Yugoslavia OR Zambia OR Zimbabwe OR Rhodesia ) |
| Intervention | - Counsel*, counseling, counseling+, - Group intervention* - Social support, emotional support, peer support, support group - Home visit* - Storytell*, stories, narration, psychoeducation, social media, health education, interview* - Focus group*” - Pilot* - Mobile phone*, smartphone*, mobile app*, mhealth, ehealth, mobile health, telemedicine, video*, online, internet, computer* - Psychosocial support, psychosocial intervention*, psychosocial wellbeing, psychotherapy |
| Control | - Standard of care - None |
| Outcome | - stigma*, shame, shame+, discriminat* , stereotyp* , prejudice* , self concept* - social withdrawal, social isolation, social exclusion, social stigma, social discrimination, social isolation+ - quality of life - depression, anxiety, stress, psychological+, mental disorders+ |

**Annex B.** Quality of the included studies

## *Quality Assessment of included study*

| **No** | **Quality Criteria** | **Yes**  **n(%)** | **No**  **n(%)** | **Unclear**  **n(%)** | **N/A**  **n(%)** |
| --- | --- | --- | --- | --- | --- |
| 1 | Were aims clearly stated? | 30 (100.0) | 0 (0.0) | 0 (0.0) | 0 (0.0) |
| 2 | Were designs appropriate for the stated objectives? | 30 (100.0) | 0 (0.0) | 0 (0.0) | 0 (0.0) |
| 3 | Was a justification for sample size given? | 20 (67.7) | 6 (20.0) | 4 (13.3) | 0 (0.0) |
| 4 | Was evidence on reliability and validity of measures provided? | 21 (70.0) | 3 (10.0) | 6 (20.0) | 0 (0) |
| 5 | Were statistics reported accurately? | 21 (70.0) | 0 (0) | 0 (0) | 9 (30.0) |
| 6 | Was the sample selection relatively unbiased? | 7 (23.3) | 22 (73.3) | 1 (3.3) | 0 (0) |
